# Association between fatty liver index and cardiometabolic multimorbidity: evidence from the cross-sectional national health and nutrition examination survey

**DOI:** 10.3389/fcvm.2024.1433807

**Published:** 2024-09-05

**Authors:** Xinsheng Gu, Di Gao, Xinjian Zhou, Yueyou Ding, Wenrui Shi, Jieun Park, Shaohui Wu, Yue He

**Affiliations:** ^1^ Department of Cardiology, Shanghai Eighth People’s Hospital, Shanghai, China; ^2^ Department of Intensive Care Unit, Shanghai Eighth People’s Hospital, Shanghai, China; ^3^ Department of Cardiology, Shanghai Chest Hospital Affiliated to Shanghai Jiao Tong University, Shanghai, China; ^4^ School of Medicine, Shanghai Jiao Tong University, Shanghai, China

**Keywords:** non-alcoholic fatty liver disease, fatty liver index, cardiometabolic multimorbidity, NHANES, general population

## Abstract

**Background:**

Metabolic dysfunction associated steatotic liver disease (MASLD) contributes to the cardiometabolic diseases through multiple mechanisms. Fatty liver index (FLI) has been formulated as a non-invasive, convenient, and cost-effective approach to estimate the degree of MASLD. The current study aims to evaluate the correlation between FLI and the prevalent cardiometabolic multimorbidity (CMM), and to assess the usefulness of FLI to improve the detection of the prevalent CMM in the general population.

**Methods:**

26,269 subjects were enrolled from the National Health and Nutrition Examination Survey 1999–2018. FLI was formulated based on triglycerides, body mass index, γ -glutamyltransferase, and waist circumference. CMM was defined as a history of 2 or more of diabetes mellitus, stroke, myocardial infarction.

**Results:**

The prevalence of CMM was 10.84%. With adjustment of demographic, anthropometric, laboratory, and medical history covariates, each standard deviation of FLI leaded to a 58.8% risk increase for the prevalent CMM. The fourth quartile of FLI had a 2.424 times risk for the prevalent CMM than the first quartile, and a trend towards higher risk was observed. Smooth curve fitting showed that the risk for prevalent CMM increased proportionally along with the elevation of FLI. Subgroup analysis demonstrated that the correlation was robust in several conventional subpopulations. Receiver-operating characteristic curve analysis revealed an incremental value of FLI for detecting prevalent CMM when adding it to conventional cardiometabolic risk factors (Area under the curve: 0.920 vs. 0.983, *P* < 0.001). Results from reclassification analysis confirmed the improvement from FLI.

**Conclusion:**

Our study demonstrated a positive, linear, and robust correlation between FLI and the prevalent CMM, and our findings implicate the potential usefulness of FLI to improve the detection of prevalent CMM in the general population.

## 1 Introduction

With the worldwide trend of aging, multimorbidity has become a burden for public health due to its association with reduced life quality, disability, and mortality (1–3). Among numerous types of multimorbidity, cardiometabolic multimorbidity (CMM), defined as a history of 2 or more of diabetes mellitus, stroke, and myocardial infarction, is the most common and hazardous type. A previous study has reported that CMM patients have a 12–15 years reduction in life expectancy at the age of 60, and a 3.7–6.9 times risk of all-cause mortality compared to people without cardiometabolic disease, and the risk increment is much higher than those with only 1 cardiometabolic disease (1). Due to this grim situation, exploring and expanding the risk factor profile of CMM, thereby improving the early identification of CMM is under great need.

Metabolic dysfunction associated steatotic liver disease (MASLD) has been proved to be closely related to cardiometabolic diseases (4–6). MASLD shares similar risk factors with cardiometabolic diseases, including lifestyle habits and metabolic dysfunction (7). A recent study based on a Swedish cohort revealed that MASLD is associated with incident major adverse cardiovascular events and cardiovascular mortality (6). Furthermore, the presence of MASLD increases the risk of developing diabetes, while diabetes also augments the risk of MASLD, the 2 diseases form notorious circle towards higher cardiometabolic risk (8). The pathophysiological mechanisms underlying the association between MASLD and cardiometabolic diseases are only partially revealed, current evidence have demonstrated that it involves endothelial dysfunction, vascular inflammation, and impaired lipid and glucose metabolism (7, 8). Based on the close relationship between MASLD and cardiometabolic diseases, monitoring of MASLD could be a possible way to refine the early identification of cardiometabolic multimorbidity in the general population. However, the current diagnosis of MASLD depending on abdominal image techniques and liver biopsy (9), which are unavailable in the primary care conditions and cannot be used for frequent monitoring. Therefore, a non-invasive, convenient, and cost-effective approach for routine monitoring of MASLD degree is needed for potential improvement of the early detection of cardiometabolic multimorbidity in the primary care conditions.

Fatty liver index (FLI) was formulated to evaluate the degree of MASLD (10). Published studies have demonstrated its efficacy in predicting several cardiovascular diseases (11–13). However, whether FLI is associated with cardiometabolic multimorbidity is still unknown. Accordingly, the current study aimed to evaluate the association between FLI and cardiometabolic multimorbidity in the general population, and estimate the value of FLI in improving the detection cardiometabolic multimorbidity in the primary care conditions.

## 2 Methods

### 2.1 Study population

The datasets used in the current analysis were obtained from the National Health and Nutrition Examination Survey (NHANES) website, covering 1999 to 2018. NHANES is an ongoing program conducted by the National Center for Health Statistics, involving a series of independent, nationally representative surveys. NHANES adopts a cross-sectional design. The survey has been conducted every two years in the United States over the past two decades. The survey employed a multistage, stratified, and clustered probability sampling design to maintain its representativeness. Data from different survey cycles can be combined for integrated analysis. Detailed information about NHANES, including recruitment procedures, population characteristics, and study design, can be found on the Centers for Disease Control and Prevention's website (https://www.cdc.gov/nchs/nhanes/index.htm).

**Figure 1:**
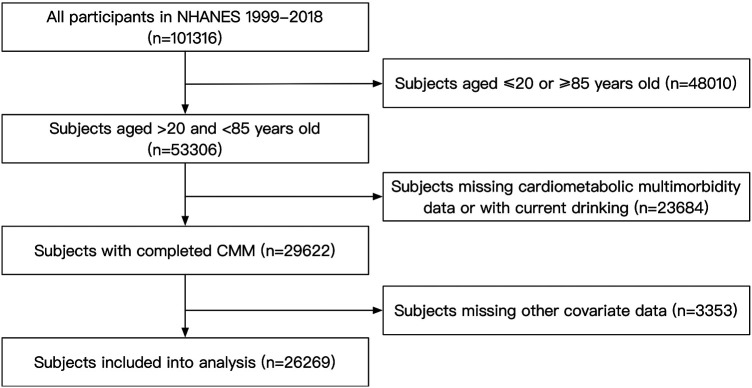
Flow chart of the subject's enrollment.

For this analysis, we included subjects aged 20–85 years who participated in NHANES from 1999 to 2018 (*n* = 101,316). The exclusion criteria were current drinking status, missing CMM data and missing covariates data. In total, our study included 26,269 participants (Figure 1). The NHANES protocol has been approved by the NCHS Institutional Ethics Review Board, our study contained no personally identifiable information. Therefore, further ethical review was not required. All the data used in our study can be accessed through the official NHANES website.

### 2.2 Data collection and measurements

Data collection involved interviews conducted in participants' homes, followed by laboratory tests at a mobile examination center. Trained staff used a computer-assisted interview system to collect the demographic data. If a participant was unable to answer a question, a family member provided a response. Socioeconomic status was assessed using the poverty-to-income ratio, which compares family income with the federal poverty threshold. Current smoking was classified as those who reported smoking cigarettes “some days” or “every day” in response to the question, “Do you currently smoke cigarettes?”. The concept of myocardial infarction and stroke were derived from the following questions: “Has a doctor or other health professional ever told you that you had myocardial infarction?”, “Has a doctor or other health professional ever told you that you had stroke?”

Anthropometric measurements were performed using a standardized procedure. Heights were measured to the nearest 0.1 cm, and weight was measured to the nearest 0.1 kg. Waist circumference (WC) was measured at the horizontal level 1 cm above the umbilicus. Blood pressure measurements were obtained after at least 5 min of quiet sitting. In this study, the mean of three blood pressure readings was analyzed. On the NHANES website, the “Physician Examination Methods Manual” provides further information regarding blood pressure measurements.

Laboratory examinations were conducted at laboratories certified by the CDC. γ-glutamyltransferase (GGT) was determined by The DxC800 using an enzymatic rate method. Fasting plasma glucose (FPG) was measured using the oxygen rate method on the modular chemistry side of Beckman DxC800. Serum creatinine (Scr) was measured using the Jaffe rate method on the DxC800 modular chemistry side. Blood lipids were quantified by an enzymatic assay using Roche Modular P and Roche Cobas 60,000 chemistry analyzer.

### 2.3 Definition

Body mass index (BMI) was calculated as weight (kg) ratio to height (m) squared. Obesity was defined as a BMI ≥30 kg/m^2^. Answering “Yes” to the question “Now taking prescribed medicine for hypertension” was determined as anti-hypertensive therapy; A mean systolic blood pressure (SBP) ≥140 mmHg, and/or a mean diastolic blood pressure (DBP) ≥90 mmHg, and/or anti-hypertensive therapy were indicated as hypertension (14). Answering “Yes” to the question “Take diabetic pills to lower blood sugar” or “Taking insulin now” was regarded as anti-diabetic therapy; FPG ≥7 mmol/L and/or self-reported use of anti-diabetic therapy was defined as diabetes (15). Low-density lipoprotein cholesterol (LDL-c) was calculated following the Friedewald formula (16). Answering “Yes” to the question “Now taking prescribed medicine for cholesterol” during the interview was defined as lipid-lowering therapy. The following standard formula calculated FLI: FLI = [e^0.953^ ^× ln(triglycerides) + 0.139 × BMI + 0.718 × ln(GGT) + 0.053 × WC − 15.745^)]/[1 + e^0.953^ ^× ln(triglycerides) + 0.139 × BMI + 0.718 × ln(GGT) + 0.053 × WC − 15.745^] × 100 (10). Cardiometabolic multimorbidity was defined as a history of 2 or more of the following: diabetes mellitus, stroke, myocardial infarction (1). The Framingham risk score was calculated according to D'Agostino's work, the score was calculated based on age, total cholesterol (TC), high-density lipoprotein cholesterol (HDL-c), SBP, smoking status, and diabetes, and the score was calculated differently based on sex and anti-hypertensive therapy (17).

### 2.4 Statistical analysis

In our study, we employed a statistical weighting to account for the survey design of NHANES. Due to the survey weighted design of NHANES, categorical variables were summarized using frequencies and 95% confidence intervals (CI), while continuous variables were presented as mean values with corresponding 95% CI (skewed variables are also presented as mean with 95% CI because the statistical software cannot calculate median with 95% CI under survey weighting). The intergroup difference for continuous variables was detected by *t*-test. Categorical variables were tested by Chi-square test. We also provided a Supplementary Table S1 to present subjects' characteristics without survey weighting, continuous variables with normal and skewed distribution were summarized as mean ± standard deviation and median (interquartile), respectively. The intergroup difference for continuous variables with normal and skewed distribution was detected by *t*-test and Mann-Whitney *U*-test, respectively. Categorical variables were presented with number (percentage) and tested by Chi-square test. The statistical analysis contained two main parts. In part one, the association between FLI and the risk for prevalent CMM was assessed using multivariate logistic regression analysis, and the results were reported as odds ratios (ORs) with 95% CIs. FLI was first regarded as a continuous variable in the regression analysis, the results were summarized as Per standard deviation (SD) change. Then, FLI was divided into quartiles and analyzed in the regression analysis as a categorical variable. Finally, a P for trend analysis would test whether the ORs for quartile 1 to quartile 4 had a statistically significant decrease trend. Additionally, we applied a generalized additive model with a spline smooth-fitting function to explore the linearity of the association across the entire range of FLI. Then, we employed subgroup analysis to test whether the main result from logistic regression was robust in several conventional subpopulations. In part two, we performed receiver-operating characteristic curve (ROC) analysis and reclassification analysis to evaluate the potential value of FLI in improving the detection of prevalent HF. The reclassification analysis included the continuous net reclassification index (NRI) and integrated discrimination index (IDI). All statistical analyses were conducted using Stata Statistical Software (version 15.0; StataCorp. LLC., College Station, TX, USA), R (The R Foundation), and EmpowerStats (X&Y Solutions, Inc., Boston, MA, USA). Statistical significance was defined as a two-tailed *P*-value less than 0.05.

## 3 Results

### 3.1 Subjects' characteristics

The characteristic data were summarized in Table 1 and Supplementary Table S1. The prevalence of CMM was 10.84% (2,848 of 26,269). As for the demographic data, CMM group had a significant higher age level (60.25years vs. 45.61years) and male percentage (52.26% vs. 48.23%). Race distribution was also different between groups, CMM group had higher percentages of Mexican American (9.30% vs. 8.00%) and black population (14.05% vs. 10.21%) while non-CMM group had a higher rate of white population (63.46% vs. 70.09%). Current smoking was slightly lower in the CMM group than the non-CMM group (15.82% vs. 17.57%, *P* = 0.048). And CMM group had a relatively lower poverty-to-income ratio level than the non-CMM group (2.66 vs. 3.05). Regarding the anthropometric parameters, CMM group had significantly lower height (167.72 cm vs. 169.09 cm), mean DBP (68.17 mmHg vs. 70.62 mmHg) levels, and higher weight (92.34 kg vs. 81.49 kg), BMI (32.69 kg/m*2 vs. 28.42 kg/m*2), WC (111.09 cm vs. 97.69 cm), mean SBP (129.39 mmHg vs. 121.33 mmHg) levels than the non-CMM group. Laboratory data showed that the levels of FPG (8.82 mmol/L vs. 5.18 mmol/L),GGT (34.12 U/L vs. 26.88 U/L) and Scr (88.27 μmol/L vs. 77.97 μmol/L) were significantly higher in CMM group, while TC (4.75 mmol/L vs. 5.09 mmol/L), LDL-c (2.46 mmol/L vs. 2.92 mmol/L), and HDL-c (1.21 mmol/L vs. 1.41 mmol/L) levels were lower in the CMM group. For the medical history data, the rates of anti-hypertensive therapy (66.02% vs. 22.09%), anti-diabetic history (79.96% vs. 6.30%), and lipid-lowering therapy (54.88% vs. 12.57%) were remarkably higher in the CMM group than in the non-CMM group. The percentages of hypertension (72.69% vs. 31.20%), diabetes (93.34% vs. 4.96%), myocardial infarction (18.63% vs. 1.93%), and stroke (13.97% vs. 1.46%) were also higher in the CMM group. Finally, FLI (75.13 vs. 50.04) was also significantly higher in the CMM group than in the non-CMM group.

**Table 1:** Sociodemographic and health characteristics of study participants with and without cardiometabolic multimorbidity (*n* = 26,269).

Variables	Total (26,269)	CMM (*n* = 2,848)	Non-CMM (*n* = 23,421)	*P*-value
Age (years)	46.77 (46.28–47.26)	60.25 (59.55–60.94)	45.61 (45.12–46.11)	<0.001
Male (%)	48.55 (48.00–49.10)	52.26 (49.98–54.53)	48.23 (47.66–48.81)	0.001
Race (%)				
Mexican American	8.11 (6.74–9.73)	9.30 (7.04–12.20)	8.00 (6.69–9.55)	<0.001
Other Hispanic	4.96 (4.13–5.96)	5.56 (5.25–7.24)	4.91 (4.09–5.90)	
Non-Hispanic white	69.56 (66.77–72.22)	63.46 (59.69–67.06)	70.09 (67.32–72.72)	
Non-Hispanic black	10.52 (9.20–12.01)	14.05 (11.94–16.46)	10.21 (8.93–11.67)	
Others	6.85 (6.17–7.59)	7.64 (6.38–9.12)	6.78 (6.09–7.53)	
Current smoking (%)	17.43 (16.55–18.36)	15.82 (14.25–17.53)	17.57 (16.65–18.53)	0.048
Poverty-to-income ratio	3.02 (2.94–3.09)	2.66 (2.56–2.76)	3.05 (2.98–3.12)	<0.001
Height (cm)	168.98 (168.78–169.19)	167.72 (167.15–168.28)	169.09 (168.89–169.29)	<0.001
Weight (kg)	82.35 (81.89–82.82)	92.34 (91.17–93.50)	81.49 (81.03–81.96)	<0.001
BMI (kg/m*2)	28.76 (28.59–28.92)	32.69 (32.28–33.09)	28.42 (28.25–28.58)	<0.001
WC (cm)	98.75 (98.32–99.19)	111.09 (110.24–111.93)	97.69 (97.26–98.12)	<0.001
SBP (mmHg)	121.97 (121.58–122.35)	129.39 (128.36–130.42)	121.33 (120.96–121.70)	<0.001
DBP (mmHg)	70.43 (70.02–70.83)	68.17 (67.53–68.81)	70.62 (70.21–71.03)	<0.001
FPG (mmol/L)	5.47 (5.44–5.50)	8.82 (8.63–9.01)	5.18 (5.17–5.20)	<0.001
TC (mmol/L)	5.07 (5.04–5.09)	4.75 (4.69–4.82)	5.09 (5.07–5.12)	<0.001
Triglycerides (mmol/L)	1.71 (1.68–1.74)	2.35 (2.24–2.48)	1.65 (1.62–1.68)	<0.001
LDL-c (mmol/L)	2.88 (2.87–2.91)	2.46 (2.40–2.52)	2.92 (2.90–2.94)	<0.001
HDL-c (mmol/L)	1.40 (1.39–1.41)	1.21 (1.19–1.24)	1.41 (1.40–1.42)	<0.001
GGT (U/L)	27.46 (26.82–28.10)	34.12 (32.12–36.11)	26.88 (26.25–27.51)	<0.001
Scr (μmol/L)	78.78 (78.31–79.26)	88.27 (73.61–76.66)	77.97 (77.50–78.43)	<0.001
Anti-hypertension therapy (%)	25.58 (24.59–26.60)	66.02 (63.46–68.48)	22.09 (21.14–23.06)	<0.001
Anti-diabetic therapy (%)	6.94 (6.53–7.36)	79.96 (77.74–82.01)	6.30 (5.10–7.71)	<0.001
Lipid-lowering therapy (%)	15.94 (15.21–16.69)	54.88 (52.21–57.53)	12.57 (11.87–13.31)	<0.001
Hypertension (%)	34.50 (33.46–35.56)	72.69 (69.98–75.25)	31.20 (30.17–32.25)	<0.001
Diabetes (%)	12.20 (11.64–12.79)	93.34 (92.00–94.47)	4.96 (4.62–5.32)	<0.001
Myocardial infarction (%)	3.26 (2.99–3.55)	18.63 (16.87–20.53)	1.93 (1.74–2.15)	<0.001
Stroke (%)	2.46 (2.25–2.68)	13.97 (12.25–15.89)	1.46 (1.31–1.63)	<0.001
Framingham risk score (%)	3.44 (3.29–3.58)	11.54 (11.13–11.95)	2.74 (.61–2.87)	<0.001
FLI	52.03 (51.20–52.85)	75.13 (73.61–76.66)	50.04 (49.22–50.85)	<0.001

Data were summarized as mean (95% confidence intervals) or numbers (95% confidence intervals) according to their data type. CMM, cardiometabolic multimorbidity; BMI, body mass index; WC, waist circumference; SBP, systolic blood pressure; DBP, diastolic blood pressure; FPG, fasting plasma glucose; TC, total cholesterol; LDL-c, low-density lipoprotein cholesterol; HDL-c, high-density lipoprotein cholesterol; GGT, γ -glutamyltransferase; Scr, serum creatinine; FLI, fatty liver index.

### 3.2 Association between FLI and prevalent CMM

**Figure 2:**
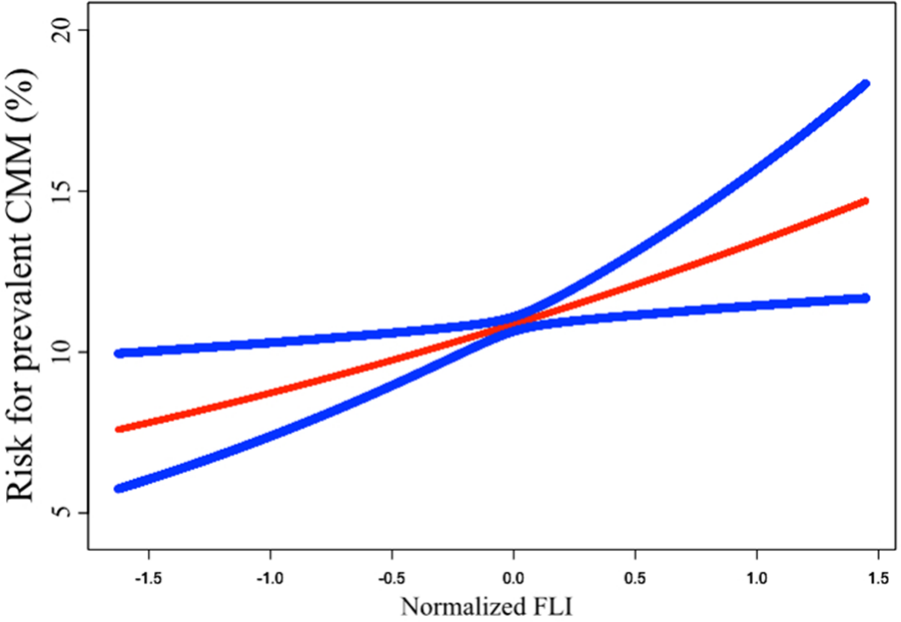
Smooth curve fitting to evaluate the linearity of the correlation between FLI and the prevalent CMM. The model was adjusted for age, sex, race, current smoking, poverty-to-income ratio, BMI, WC, Scr, FPG, TC, HDL-c, SBP, anti-hypertensive therapy, anti-diabetic therapy, and lipid-lowering therapy (The same as Model 2 in Table 2). The blue lines depicted the pointwise 95% CI, and the red line showed the estimated risk of prevalent CMM. The association is linear in the whole range of FLI. FLI, fatty liver index; CMM, cardiometabolic multimorbidity; BMI, body mass index; WC, waist circumference; Scr, serum creatinine; FPG, fasting plasma glucose; TC, total cholesterol; HDL-c, high-density lipoprotein cholesterol; SBP, systolic blood pressure; CI, confidence intervals.

The results of the Logistic regression analysis were showed in Table 2. When analyzed as a continuous variable, each SD increase of FLI could cast a 2.544 times risk increment. After adjustment of age, sex, race, current smoking, and poverty-to-income ratio, the risk increase shrank to 2.035 times for each SD increase of FLI. Further adjustment of BMI, WC, Scr, FPG, TC, HDL-c, SBP, anti-hypertensive therapy, anti-diabetic therapy, and lipid-lowering therapy diminished the risk to 1.588 times. When dividing FLI into quartiles, the top quartile showed a 2.424 times risk of prevalent CMM than the bottom quartile in Model 2, and the data demonstrated a trend of increasing risk from quartile 1 to quartile 4 (P for trend = 0.001). To confirm the trend of increasing risk that observed in Logistic regression analysis, we conducted a smooth curve fitting analysis (Figure 2). The result showed that risk for prevalent CMM increased linearly along with the elevation of FLI in the whole range of FLI.

**Table 2:** Association between FLI and the risk of prevalent CMM.

Variables	Odds ratio (95% CI)					
	Crude	*P*-value	Model 1	*P*-value	Model 2	*P*-value
FLI (Per SD increase)	2.544 (2.366–2.736)	<0.001	2.035 (2.020–2.368)	<0.001	1.588 (1.214–2.077)	0.001
Quartiles of FLI						
Quartile 1	Reference		Reference		Reference	
Quartile 2	2.622 (1.887–3.642)	<0.001	1.685 (1.192–2.381)	<0.001	1.059 (0.553–2.029)	0.862
Quartile 3	6.516 (4.847–8.759)	<0.001	3.781 (2.776–5.151)	<0.001	1.634 (0.862–3.099)	0.131
Quartile 4	14.703 (10.546–20.497)	<0.001	10.083 (7.110–14.300)	<0.001	2.424 (1.190–4.939)	0.015
P for trend		<0.001		<0.001		0.001

Crude model: no adjustment.

Model 1: age, sex, race, current smoking, poverty-to-income ratio.

Model 2: Model 1+ BMI, WC, Scr, FPG, TC, HDL-c, SBP, anti-hypertensive therapy, anti-diabetic therapy, lipid-lowering therapy.

FLI, fatty liver index; CMM, cardiometabolic multimorbidity; SD, standard deviation; BMI, body mass index; WC, waist circumference; Scr, serum creatinine; FPG, fasting plasma glucose; TC, total cholesterol; HDL-c, high-density lipoprotein cholesterol; SBP, systolic blood pressure.

### 3.3 Association between FLI and Framingham risk score

Supplementary Figure S1 depicted the association between FLI and Framingham risk score. The plot showed that FLI had a weak but significant and positive correlation with the Framingham risk score (R^2 ^= 0.219, *P* < 0.001). The risk increased along with the elevation of the FLI value.

### 3.4 Subgroup analysis

**Figure 3:**
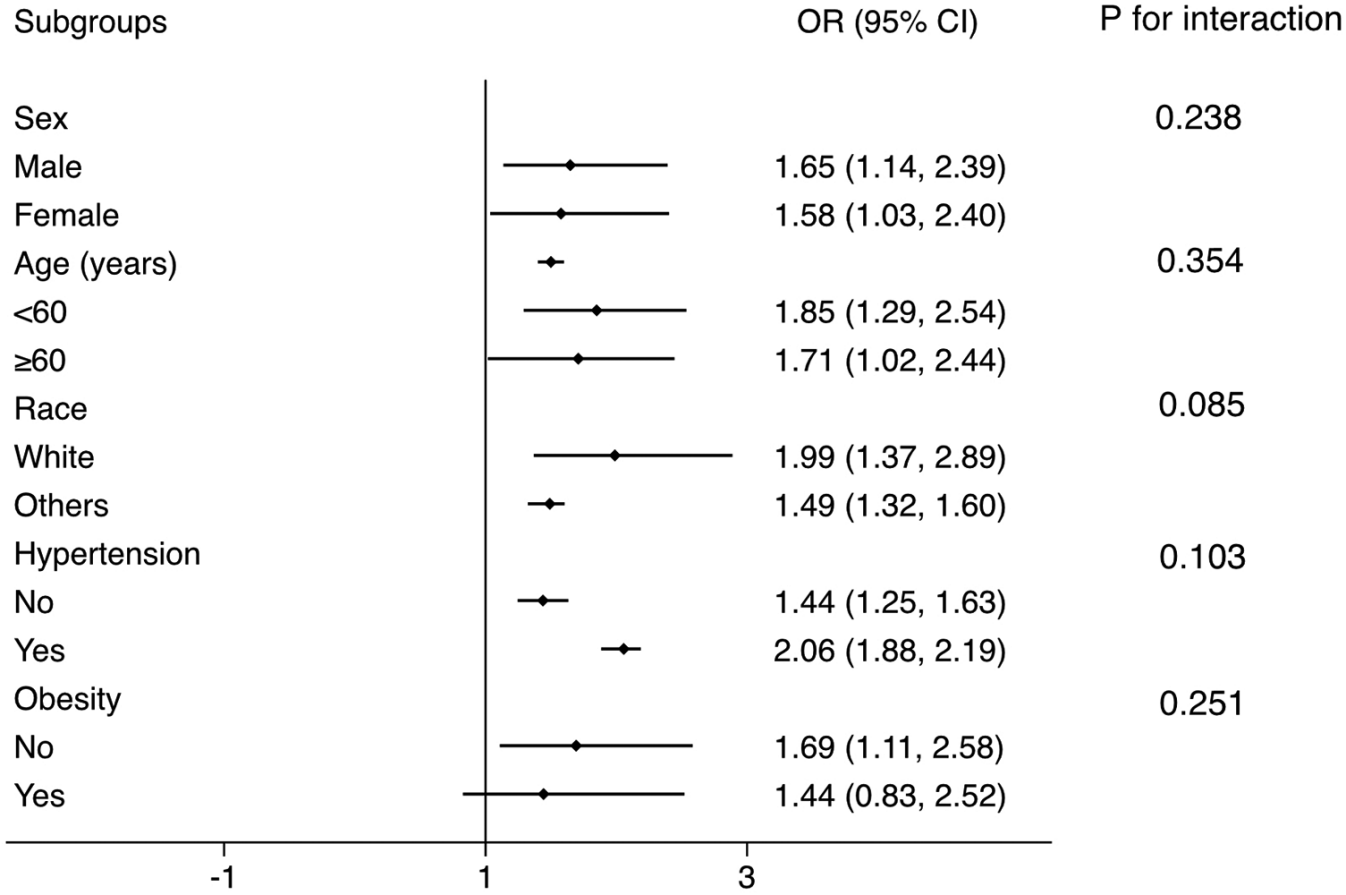
Subgroup analysis for the correlation between FLI and the prevalent CMM. The multivariate logistic model adjusted for all variables used in Model 2 of Table 2, except for the variable used to define subgroups. The association was robust to age, sex, race, hypertension, and obesity subgroups. FLI, fatty liver index; CMM, cardiometabolic multimorbidity.

Subgroup analysis was conducted to test whether the results from the whole population was robust in several conventional subpopulations (Figure 3). The logistic regressions were adjusted for all covariates in the Model 2 of Logistic regression analysis, except for those used to define subgroups. The results showed that the association was robust in sex, age, race, hypertension, and obesity subgroups (all P for interaction >0.05).

### 3.5 Value of FLI to improve the detection of prevalent CMM

ROC and reclassification analyses were performed to estimate the value of FLI to improve the detection of prevalent CMM (Table 3). In ROC analysis, the area under the curve (AUC) of FLI alone was 0.699 (95% CI: 0.693–0.704). When adding eGDR into clinical risk factors, eGDR significantly improved the AUC (0.920 vs. 0.983, P for comparison <0.001). In the reclassification analysis, both continuous NRI (0.326, 95% CI: 0.288–0.363, *P* < 0.001) and IDI (0.021, 95% CI: 0.017–0.023, *P* < 0.001) confirmed a significant enhancement from FLI to improve the detection of prevalent CMM.

**Table 3:** ROC and reclassification analysis for FLI to improve the detection of prevalent CMM.

Model	AUC (95% CI)	*P*-value	P for comparison	NRI (continuous)	*P*-value	IDI	*P*-value
FLI	0.699 (0.693–0.704)	<0.001	–	–	–	–	–
Clinical risk factors^a^	0.920 (0.916–0.923)	<0.001	<0.001^b^	–	–	–	–
Clinical risk factors + FLI	0.983 (0.981–0.984)	<0.001	<0.001^c^	0.326 (0.288–0.363)	<0.001	0.021 (0.017–0.023)	<0.001

ROC, receiver operating curve; FLI, fatty liver index; CMM, cardiometabolic multimorbidity; AUC, area under the curve; NRI, net reclassification index; IDI, integrated discrimination index; BMI, body mass index; WC, waist circumference; Scr, serum creatinine; FPG, fasting plasma glucose; TC, total cholesterol; HDL-c, high-density lipoprotein cholesterol; SBP, systolic blood pressure.

^a^ Clinical risk factors: age, sex, race, current smoking, poverty-to-income ratio, BMI, WC, Scr, FPG, TC, HDL-c, SBP, anti-hypertensive therapy, anti-diabetic therapy, and lipid-lowering therapy.

^b^ Comparison of the AUC between FLI alone and clinical risk factors.

^c^ Comparison of the AUC between clinical risk factors and clinical risk factors + FLI.

## 4 Discussion

Our study provides two major findings: 1. There is a significant and negative association between FLI, a surrogate of MASLD severity, and the risk of prevalent CMM in the general population. Furthermore, the association is linear in the whole range of FLI. Moreover, the association is robust among conventional cardiovascular sub-populations. 2. FLI could improve the detection of prevalent CMM in the general population. Our findings provide an evidence for the potential usefulness of FLI to improve the detection of prevalent CMM in the general population, especially in primary care conditions.

In part one of the current analysis, the logistic regression revealed a significant and negative association between FLI and prevalent CMM after adjusting common demographic, laboratory, anthropometric, and medical history data. Hence, FLI could be independently associated with the risk for prevalent CMM in the general population. In the quartile analysis, the risk of prevalent CMM continuously increased from quartile 1 to quartile 4, and the P for trend <0.001. Smooth curve fitting was conducted to confirm this linearity. The results showed that the risk for prevalent CMM elevated proportionally with the increase of FLI (Figure 2). Based on the findings from the logistic regression and smooth curve fitting, FLI could serve as a linear indicator of the risk for prevalent CMM in the general population.

Then we tested whether our main results from the whole population was robust to conventional subpopulations. In sex, age, race, and hypertension subgroups, the OR value was consistent and similar to the OR in the whole population. All the P for interaction >0.05, which means there is no interaction between these subgrouping variates and the association between FLI and prevalent CMM, and applying the results derived from the general population to these subpopulations is reasonable.

Based on the ROC and reclassification analysis results, FLI could improve the detection of prevalent CMM in the general population. Although the AUC of eGDR alone was limited, a significant improvement was achieved when adding FLI to conventional cardiovascular risk factors (P for comparison <0.001), implicating the incremental value of FLI to improve the detection of prevalent CMM. Furthermore, Because the AUC of FLI alone is significantly lower than that of existing conventional cardiometabolic risk factors (P for comparison <0.001), the value of FLI is more prominent when adding it to conventional risk factors than using FLI alone. However, although ROC analysis is the most common approach to evaluate the value of a novel marker, it still has its limitations. ROC analysis focus on the comparison of the diagnostic ability of different models rather than evaluating the value of a novel marker to optimize the diagnostic ability of the whole model (18). Thus, the sensitivity of ROC analysis to assess the value of a novel index in improving the detection of prevalent diseases is relatively low (19). To address the drawback of ROC analysis, reclassification analysis has been proposed, intending to evaluate the improvement from novel indexes for refining the detection of prevalent diseases (20–22). Compared with ROC analysis, reclassification analysis focused on the incremental value of a novel biomarker for diagnosing or predicting diseases rather than the ability of the whole diagnosis or prediction model. Therefore, reclassification analysis could specifically test the diagnostic or predictive value of the novel biomarkers. However, reclassification analysis also has its limitations. First, it could not compare the diagnostic or predictive value of the two models. Therefore, the readers could not acquire the overall improvement of the diagnostic or predictive value of the new model containing the novel biomarker. Second, reclassification analysis is rarely used in studies, and the basic model used in different studies is variant. Therefore, comparing NRI and IDI of different biomarkers from different studies is impractical. Accordingly, the significance of NRI and IDI is more important than their values. Third, the reclassification analysis has a relatively higher sensitivity than the ROC analysis. Hence, some biomarkers could be overestimated by reclassification analysis. Overall, ROC and reclassification analysis evaluate a novel biomarker from different angles. The two analyses have their advantages and disadvantages. Since they are complementary, the results of the two analyses should be discussed together. In the current work, both continuous NRI and IDI confirmed the significant improvement from FLI to improve the detection of prevalent CMM. In summary, both reclassification and ROC analysis suggest the potential usefulness of FLI to improve the detection of prevalent CMM in the general population.

The results in Table 1 showed that the CMM group had a relatively lower LDL-c and TC level than the non-CMM group. Considering the CMM group had a remarkably higher rate of receiving lipid-lowering therapy than the non-CMM group (54.88% vs. 12.57%), this phenomenon is still reasonable. Furthermore, we re-conducted the logistic regression by adding LDL-c to the covariates (Supplementary Table S2). The results showed that the OR for LDL-c was insignificant (1.005, 95% CI: .873–1.157, *P* = 0.942), while the ORs for TC (0.801, 95% CI: 0.689–0.932, *P* = 0.004), HDL-c (0.581, 95% CI: 0.393–0.860, *P* = 0.007), and lipid-lowering therapy (1.564, 95% CI: 1.183–2.068, *P* < 0.001) were still significant. Therefore, our results suggest that the increased CMM risk should be attributed to the reduced HDL-c, but not the increased LDL-c. Accordingly, we decided not to add LDL-c as a covariate in the main logistic models to avoid multicollinearity of the covariates.

Diabetes, one of the elements of CMM, is used to calculate the Framingham risk score. Furthermore, the other two elements of CMM, namely myocardial infarction and stroke, were the target outcomes of Framingham risk score. Therefore, it is reasonable that the risk of CMM increased along with the elevation of Framingham risk score value. The severity of MASLD, which is estimated by FLI, creates chronic inflammation, dyslipidemia, insulin resistance, and other disorders of the internal environment, and finally leads to the development of atherosclerotic diseases (23, 24), which are target outcomes of Framingham risk score. Therefore, it is reasonable to conclude that the increment of FLI promotes the elevation of Framingham risk score, facilitates the formation of atherosclerotic diseases, and thereby boost the risk of CMM.

Studies have indicated that MASLD can be screened using the Fibrosis Index Based on 4 Factors (FIB-4), which generates a single score by integrating patient age with measurements of three biomarkers: aspartate aminotransferase, alanine aminotransferase, and platelet count (25, 26). This indicator reflects liver inflammation or damage and advanced liver fibrosis by assessing liver enzymes, age, and platelet count. FIB-4 is particularly valuable for identifying advanced liver fibrosis in the later stages of MASLD. In contrast, the FLI is effective in detecting earlier stages of liver steatosis in MASLD. The ability of FLI to identify liver steatosis at an earlier stage can facilitate timely intervention, potentially halting the progression to more severe liver disease. The relationship between FLI and FIB-4 is complementary. FLI serves as an initial screening tool to detect early liver fat accumulation, while FIB-4 can be utilized to assess the risk of progression to more advanced fibrosis in patients already identified with liver steatosis. By using these tools in tandem, clinicians can implement a more comprehensive approach to screening and managing MASLD.

The underlying mechanisms linking MASLD and the risk of cardiometabolic diseases are complicated (8, 27). First, MASLD leads to endothelial dysfunction (28). Several markers of endothelial dysfunction, including AMDA and fetuin-a, are elevated in MASLD (29–31). Endothelial dysfunction and disruption could escalate atherogenesis and subsequent cardiovascular diseases. Second, MASLD also increased the serum homocysteine level (32). Disruption of homocysteine metabolism boosts the oxidative stress, thereby triggering the pathophysiological progress of cardiovascular diseases (33). Fourth, cytokines, hepatokines, and adipokines, released by the liver during MASLD could damage the cardiovascular system (34, 35). Fifth, lipid metabolism is also changed during MASLD. HDL-c level is decreased while triglycerides and LDL-c levels are increased. Then the lipid profile changed to a more atherogenic pattern (36, 37). Last, the accumulation of lipid in liver is associated with hepatic, adipose tissue, and muscle insulin resistance, thereby increasing the risk of type 2 diabetes (38, 39).

The major clinical implication of the current study is that it described a detailed association between FLI and the prevalent CMM, thereby strengthening the association between MASLD and the prevalent CMM. FLI, and the severity of MASLD, is linearly associated with the risk for prevalent CMM, implicating that controlling of MASLD could be beneficial for prevention of CMM. Another implication of the current study is that FLI could be used to improve the early detection of prevalent CMM, especially in primary care conditions. CMM is one of the most common and hazardous multimorbidity, and it has been proofed to be associated with reduced life expectancy and increased mortality. Therefore, CMM is a status of high cardiovascular risk, especially in the elder population. However, the danger of CMM is always be ignored, especially in the primary care conditions. Accordingly, detection of CMM condition is important in cardiovascular prevention. In rural and developing regions, where access to advanced diagnostic tools and specialized care is often limited, the FLI provides a non-invasive, straightforward, and cost-effective method for assessing the risk of CMM. This approach enables early detection and intervention, which are essential for preventing complications associated with cardiometabolic diseases. Integrating FLI into routine screening protocols allows primary healthcare providers to identify high-risk individuals at an early stage. Consequently, this can prompt timely lifestyle interventions, including dietary modifications, increased physical activity, and appropriate medication management, thereby potentially reducing the incidence and severity of diseases such as type 2 diabetes, cardiovascular disease, and hypertension. Moreover, the adoption of FLI in primary care settings can guide resource allocation and inform health policy decisions. By identifying populations at elevated risk for CMM, public health officials can more effectively allocate resources, plan community health programs, and target interventions to alleviate the burden of cardiometabolic diseases. This is particularly critical in resource-limited settings where healthcare budgets are constrained. Preventing the progression of these conditions can substantially reduce healthcare costs associated with managing advanced stages and complications of these diseases.

In addition to the findings presented, this study paves the way for several avenues of further research. Future investigations should employ longitudinal designs to establish causal relationships between the FLI and CMM, addressing the limitation of causal inference inherent in the cross-sectional nature of our study. Additionally, examining the role of supplementary biomarkers and their potential synergistic interactions with FLI could offer a more comprehensive understanding of the mechanisms connecting MASLD to cardiometabolic conditions. Extending this research to diverse populations beyond the United States is essential for verifying the generalizability of our findings and for elucidating regional variations in the prevalence and risk factors of MASLD and CMM. Furthermore, developing and validating interventions aimed at reducing MASLD, including lifestyle modifications and pharmacological treatments, and assessing their impact on CMM incidence, represent critical next steps. Finally, the integration of FLI into clinical practice and the evaluation of its effectiveness in enhancing the early detection and management of CMM across various healthcare settings, particularly in primary care, could significantly improve personalized patient care and outcomes.

Our study has several limitations. First, the cross-sectional design of NHANES made us unable to determine whether there is a causal relationship between FLI and CMM. Therefore, we could not explore the value of FLI in predicting the incidence of CMM in the current study. Future studies with a longitudinal design are needed to expand our findings. Second, relying on self-reported information in NHANES raises concerns regarding recall limitations and subjectivity, potentially leading to inaccurate data. Studies with more reliable definitions are needed to confirm our conclusions. Thirdly, because our study excluded participants from NHANES who lacked relevant variables, selection bias may exist in our work. Fourth, since NHANES was only conducted in the United States, whether our findings is applicable to other populations remains unknown, Therefore, more studies containing different populations are needed to verify our results. Last, although our analysis adjusted a series of covariates, some unincluded confounders could also introduce bias into our results. Therefore, studies with more detailed information collection are also needed to confirm our results.

## Data Availability

The datasets presented in this study can be found in online repositories. The names of the repository/repositories and accession number(s) can be found below: https://wwwn.cdc.gov/nchs/nhanes/ContinuousNhanes/Default.aspx?BeginYear=1999.
