# Supplementary material for: Association between fatty liver index and cardiometabolic multimorbidity: evidence from the cross-sectional national health and nutrition examination survey
Source: Front Cardiovasc Med. 2024 Sep 5;11:1433807. doi: 10.3389/fcvm.2024.1433807 (PMC11411361; doi:10.3389/fcvm.2024.1433807)
Supplement: Supplementary file 1 [file Datasheet1.docx]

Table S1. Sociodemographic and health characteristics of study participants with and without cardiometabolic multimorbidity (n=26269) without weighting.

| Variables | Total (26269) | CMM (n=2848) | Non-CMM (n=23421) | P value |
| --- | --- | --- | --- | --- |
| Age (years) | 48.62 ± 17.84 | 62.03 ± 12.80 | 46.99 ± 17.68 | <0.001 |
| Male (%) | 12771 (48.62) | 1512 (53.09) | 11259 (48.07) | 0.001 |
| Race (%) |  |  |  | <0.001 |
| Mexican American | 4281 (16.30) | 576 (20.22) | 3705 (15.82) |  |
| Other hispanic | 2128 (8.10) | 265 (9.30) | 1863 (7.95) |  |
| Non-hispanic white | 11926 (45.40) | 1069 (37.54) | 10857 (46.36) |  |
| Non-hispanic black | 5325 (20.27) | 687 (24.12) | 4638 (19.80) |  |
| Others | 2609 (9.93) | 251 (8.81) | 2358 (10.07) |  |
| Current smoking (%) | 4479 (17.05) | 420 (14.75) | 4059 (17.33) | 0.001 |
| Poverty-to-income ratio | 2.53 ± 1.63 | 2.22 ± 1.50 | 2.57 ± 1.64 | <0.001 |
| Height (cm) | 167.44 ± 10.12 | 166.45 ± 10.26 | 167.56 ± 10.10 | <0.001 |
| Weight (kg) | 81.40 ± 20.96 | 89.16 ± 23.06 | 80.46 ± 20.49 | <0.001 |
| BMI (kg/m*2) | 28.95 ± 6.70 | 32.04 ± 7.16 | 28.58 ± 6.54 | <0.001 |
| WC (cm) | 99 ± 16.12 | 109.27 ± 16.00 | 97.81 ± 15.68 | <0.001 |
| SBP (mmHg) | 123.73 ± 18.64 | 131.48 ± 20.22 | 122.79 ± 18.21 | <0.001 |
| DBP (mmHg) | 69.71 ± 12.94 | 67.67 ± 14.64 | 69.96 ± 12.70 | <0.001 |
| FPG (mmol/L) | 5.16 (4.72-5.77) | 7.88 (6.11-10.71) | 5.05 (4.66-5.55) | <0.001 |
| TC (mmol/L) | 5.04 ±1.10 | 4.76 ± 1.24 | 5.08 ± 1.07 | <0.001 |
| Triglycerides (mmol/L) | 1.34 (0.89-2.09) | 1.74 (1.16-2.68) | 1.30 (0.87-2.01) | <0.001 |
| LDL-c (mmol/L) | 2.87 ± 0.97 | 2.49 ± 1.08 | 2.91 ± 0.95 | <0.001 |
| HDL-C (mmol/L) | 1.39 ± 0.42 | 1.24 ± 0.36 | 1.40 ± 0.43 | <0.001 |
| GGT (U/L) | 19.00 (14.00-30.00) | 23.00 (17.00-37.00) | 19.00 (14.00-29.00) | 0.002 |
| Scr (μmol/L) | 76.02 (62.76-88.4) | 80.00 (65.42-100.78) | 75.14 (61.88-88.4) | <0.001 |
| Anti-hypertension therapy (%) | 7683 (29.25) | 1930 (67.77) | 5753 (24.56) | <0.001 |
| Anti-diabetic therapy (%) | 2477 (9.43) | 2294 (80.55) | 183 (0.78) | <0.001 |
| Lipid-lowering therapy (%) | 4540 (17.28) | 1510 (53.02) | 3030 (12.94) | <0.001 |
| Hypertension (%) | 10259 (39.05) | 2121 (74.47) | 8138 (34.75) | <0.001 |
| Diabetes (%) | 3746 (14.26) | 2672 (93.82) | 1074 (4.59) | <0.001 |
| Myocardial infarction (%) | 1076 (4.10) | 519 (18.22) | 557 (2.38) | <0.001 |
| Stroke (%) | 871 (3.32) | 419 (14.71) | 452 (1.93) | <0.001 |
| Framingham risk score (%) | 0 (0-7.99) | 11.84 (7.35-17.99) | 0 (0-5.94) | <0.001 |
| FLI | 53.18 ± 32.36 | 72.65 ± 26.25 | 50.82 ± 32.23 | <0.001 |

Data were summarized as mean ± standard deviation, median (interquartile) or number (percentage) according to their data type. The intergroup difference for continuous variables with normal and skewed distribution was detected by t-test and Mann-Whitney U test, respectively. Categorical variables were tested by Chi-square test.

Abbreviations: CMM: cardiometabolic multimorbidity; BMI: body mass index; WC: waist circumference; SBP: systolic blood pressure; DBP: diastolic blood pressure; FPG: fasting plasma glucose; TC: total cholesterol; LDL-c: low-density lipoprotein cholesterol; HDL-c: high-density lipoprotein cholesterol; GGT: γ -glutamyltransferase; Scr: serum creatinine; FLI: fatty liver index.

Table S2. Re-conduction of logistic regression analysis by adding LDL-c to covariates.

| Variates | OR (95% CI) | 95% CI | P value |
| --- | --- | --- | --- |
| FLI (Per SD change) | 1.375 | 1.048-1.804 | 0.022 |
| age | 1.044 | 1.034-1.054 | <0.001 |
| sex | 0.918 | 0.706-1.193 | 0.516 |
| race | 1.12 | 1.001-1.255 | 0.049 |
| Current smoking | 2.075 | 1.531-2.812 | <0.001 |
| PIR | 0.847 | 0.786-0.912 | <0.001 |
| BMI | 1.984 | 1.932-2.038 | <0.001 |
| WC | 1.504 | 1.482-1.526 | 0.002 |
| FPG (mmol/L) | 1.746 | 1.642-1.857 | <0.001 |
| TC (mmol/L) | 0.801 | 0.689-0.932 | 0.004 |
| LDL-c (mmol/L) | 1.005 | 0.873-1.157 | 0.942 |
| HDL-c (mmol/L) | 0.581 | 0.393-0.860 | 0.007 |
| Scr (μmol/L) | 1.21 | 1.107-1.311 | 0.038 |
| SBP (mmHg) | 0.995 | 0.988-1.003 | 0.211 |
| Anti-hypertensive therapy | 1.83 | 1.369-2.447 | <0.001 |
| Anti-diabetic therapy | 2.809 | 2.072-3.808 | <0.001 |
| Lipid-lowering therapy | 1.564 | 1.183-2.068 | 0.022 |

The outcome of the multivariate logistic regression was cardiometabolic multimorbidity.

Abbreviations: OR: Odds ratio; CI: confidence interval; FLI: fatty liver index; PIR: poverty-income ratio; BMI: body mass index; WC: waist circumference; LDL-c: low-density lipoprotein cholesterol; HDL-c: high-density lipoprotein cholesterol; Scr: serum creatinine; SBP: systolic blood pressure.

Figure S1. Association between FLI and Framingham risk score.

The blue dots represent the Framingham risk score for each subject. The black solid line is the linear fitting line. FLI showed a weak but significant and positive association between FLI and the Framingham risk score, with a R2=0.219, P < 0.001.
