# Supplementary figures and images for: Association between fatty liver index and cardiometabolic multimorbidity: evidence from the cross-sectional national health and nutrition examination survey
Source: Front Cardiovasc Med. 2024 Sep 5;11:1433807. doi: 10.3389/fcvm.2024.1433807 (PMC11411361; doi:10.3389/fcvm.2024.1433807)

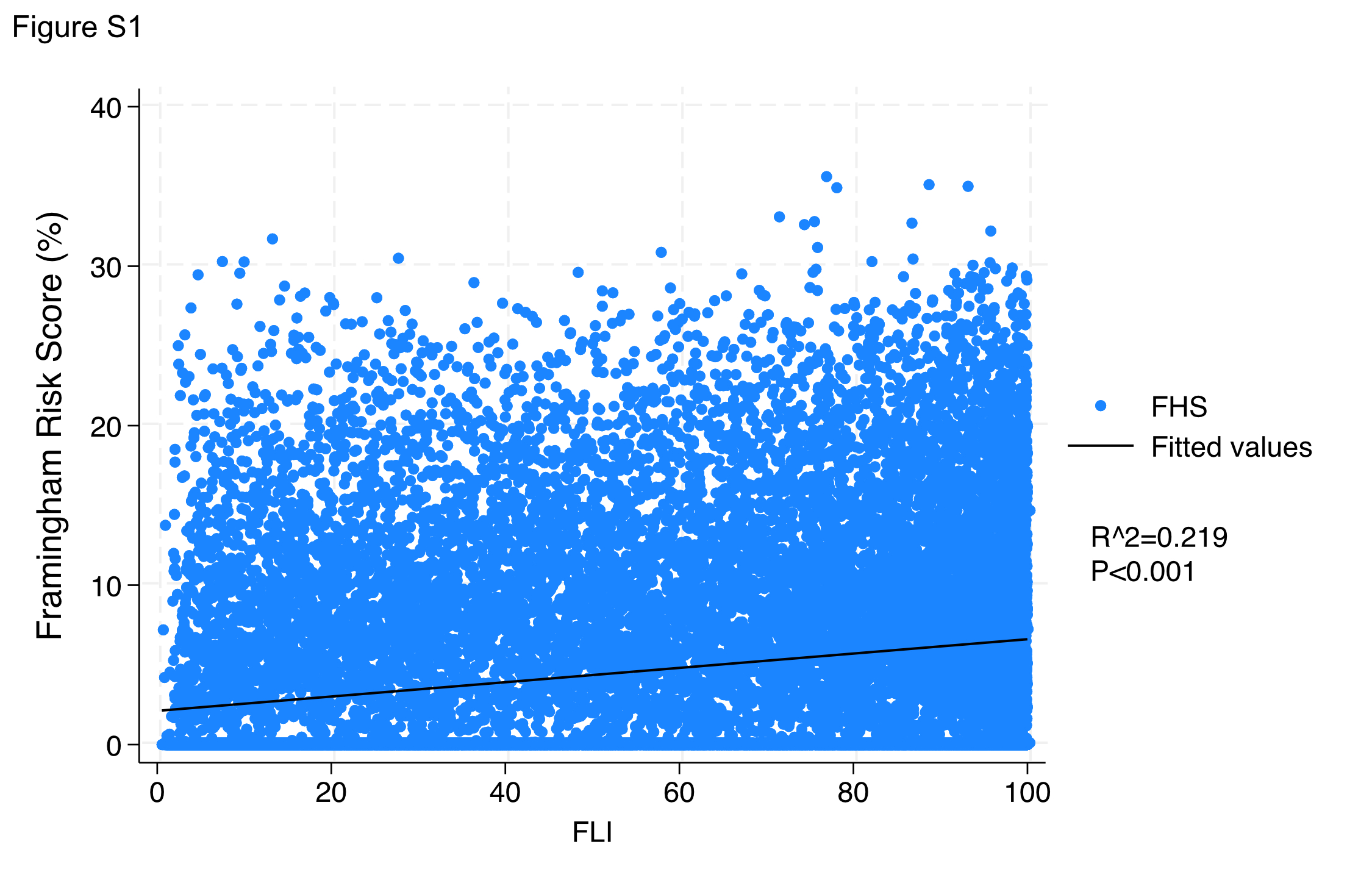

Supplement: Supplementary file 2 [file Image1.jpeg]
